# Effects of Fungicides on Rat's Neurosteroid Synthetic Enzymes

**DOI:** 10.1155/2017/5829756

**Published:** 2017-07-24

**Authors:** Xiuwei Shen, Fan Chen, Lanlan Chen, Ying Su, Ping Huang, Ren-Shan Ge

**Affiliations:** ^1^Department of Pharmacy, The Third Affiliated Hospital of Wenzhou Medical University, Wenzhou, Zhejiang 325200, China; ^2^Department of Anesthesiology, The Second Affiliated Hospital and Yuying Children's Hospital of Wenzhou Medical University, Wenzhou, Zhejiang 325027, China; ^3^Department of Pharmacy, Zhejiang Cancer Hospital, Hangzhou, Zhejiang 310022, China

## Abstract

Exposure to environmental endocrine disruptors may interfere with nervous system's activity. Fungicides such as tebuconazole, triadimefon, and vinclozolin have antifungal activities and are used to prevent fungal infections in agricultural plants. In the present study, we studied effects of tebuconazole, triadimefon, and vinclozolin on rat's neurosteroidogenic 5*α*-reductase 1 (5*α*-Red1), 3*α*-hydroxysteroid dehydrogenase (3*α*-HSD), and retinol dehydrogenase 2 (RDH2). Rat's 5*α*-Red1, 3*α*-HSD, and RDH2 were cloned and expressed in COS-1 cells, and effects of these fungicides on them were measured. Tebuconazole and triadimefon competitively inhibited 5*α*-Red1, with IC_50_ values of 8.670 ± 0.771 × 10^−6^ M and 17.390 ± 0.079 × 10^−6^ M, respectively, while vinclozolin did not inhibit the enzyme at 100 × 10^−6^ M. Triadimefon competitively inhibited 3*α*-HSD, with IC_50_ value of 26.493 ± 0.076 × 10^−6^ M. Tebuconazole and vinclozolin weakly inhibited 3*α*-HSD, with IC_50_ values about 100 × 10^−6^ M, while vinclozolin did not inhibit the enzyme even at 100 × 10^−6^ M. Tebuconazole and triadimefon weakly inhibited RDH2 with IC_50_ values over 100 × 10^−6^ M and vinclozolin did not inhibit this enzyme at 100 × 10^−6^ M. Docking study showed that tebuconazole, triadimefon, and vinclozolin bound to the steroid-binding pocket of 3*α*-HSD. In conclusion, triadimefon potently inhibited rat's neurosteroidogenic enzymes, 5*α*-Red1 and 3*α*-HSD.

## 1. Introduction

**Scheme 1:**
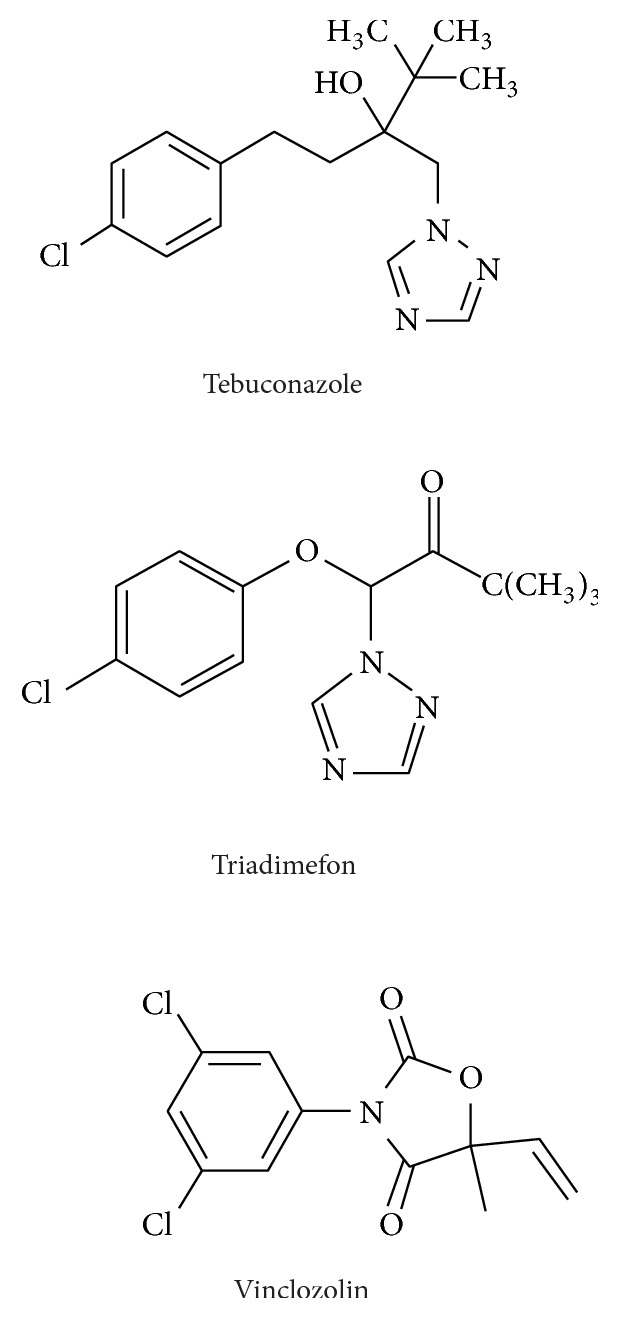
The chemical structure of fungicides.

Exposure to environmental endocrine disruptors may interfere with nervous system's activity. Fungicides such as tebuconazole (TEB) [1], triadimefon (TRI) [2], and vinclozolin (VCZ) [3] have a wide range of antifungal activities and are used to prevent fungal infections for agricultural plants. Therefore, exposure to these chemicals is very common. These fungicides contain at least one triazole or imidazole moiety in the chemical structure (Scheme 1). It is believed that these fungicides block the synthesis of fungus steroid, ergosterol. Ergosterol is a membrane component, and, therefore, these chemicals can disrupt cell membrane assembly of fungi to kill the fungi [4].

These fungicides may interfere with the steroid biosynthesis in mammals. For example, azole fungicides reduce the estrogen production via blocking aromatase [5, 6]. Neurosteroids are another set of steroids which have neurological activity [7]. These neurosteroids include allopregnanolone (ALLO) and 5*α*-androstane-3*α*, 17*β*-diol (DIOL) [7]. Although the classic steroids such as progesterone, estrogen, and testosterone act via binding to their respective nuclear receptors (progesterone, estrogen, and androgen receptors), neurosteroids allosterically activate the membrane GABA-A receptors and potentiate the central inhibition, causing anxiolytic, anticonvulsant, analgesic, and sedative effects [7, 8]. GABA-A receptors are widely present in the nervous system to exert inhibitory action on nerve activity [7].

**Scheme 2:**
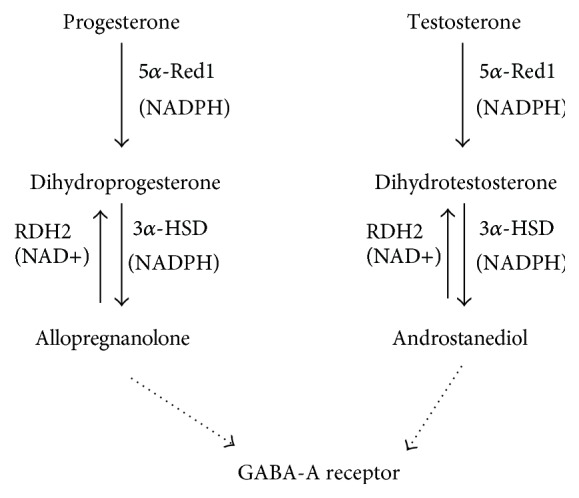
The biosynthesis and metabolism of neurosteroids, allopregnanolone and androstanediol, by three distinct enzymes: NADPH-dependent 5*α*-reductase 1 (SRD5A1), NADPH-dependent cytosolic 3*α*-hydroxysteroid dehydrogenase (AKR1C14), and NAD^+^- dependent microsomal retinol dehydrogenase 2 (RDH2).

ALLO and DIOL biosynthesis requires brain 5*α*-reductase 1 (5*α*-Red1) and 3*α*-hydroxysteroid dehydrogenase (3*α*-HSD). 5*α*-Red1 is a smooth endoplasmic reticulum NADPH-dependent enzyme [9], catalyzing progesterone or testosterone into dihydroprogesterone and dihydrotestosterone, respectively [10] (Scheme 2). 3*α*-HSD, a cytosolic enzyme, catalyzes these two steroids into ALLO or DIOL, respectively [11]. In rat's brain, microsomal NAD^+^-dependent retinol dehydrogenase 2 (RDH2) catalyzes the reverse reaction of ALLO or DIOL back to dihydroprogesterone and dihydrotestosterone, thus controlling the levels of these neurosteroids [12] (Scheme 2). Therefore, in the present study, we examined their direct effects on these neurosteroidogenic enzymes and their differential sensitivity.

## 2. Experimental Procedures

### 2.1. Chemicals

[^3^H]Testosterone, [^3^H] dihydrotestosterone, and [^3^H] DIOL were obtained from DuPont-New England Nuclear (Boston, MA). Testosterone, dihydrotestosterone, and DIOL were purchased from Steraloids (Newport, RI). TEB, TRI, and VCZ were purchased from Sigma-Aldrich (St. Louis, MO). TEB, TRI, and VCZ were dissolved in DMSO, which is used as a vehicle. Rat 3*α*-HSD gene* Akr1c14* in the expression vector pRc/CMV was a gift from Penning T. M. (University of Pennsylvania, Philadelphia, Pennsylvania). Rat's 5*α*-Red1 gene* Srd5a1 *and RDH2 gene* Rdh2* in the expression vector pcDNA1.1 were constructed previously [13]. COS-1 cell line was purchased from ATCC (Manassas, VA).

### 2.2. Transient Transfection

COS-1 cells were maintained in DMEM medium (Life Technologies, Inc., Gaithersburg, MD) supplemented with 10% fetal calf serum and 5% CO_2_ at 37°C. For transfection, 1 × 10^6^ cells were seeded per well in a six-well plate and cultured for 24 h in media supplemented with charcoal-stripped fetal calf serum to obtain 50–80% confluence. Transfection was performed by the FuGENE 6 Transfection Reagent (Roche Molecular Biochemicals, Indianapolis, IN) according to the manufacturer's protocol. 1 *µ*g DNA per well showed maximal efficiency and, therefore, this quantity was used in the transfection assays.

### 2.3. Preparation of 5*α*-Red1, 3*α*-HSD, and RDH2 Proteins

Twenty-four hours after transfection, the COS-1 cells were scraped from dishes and were homogenized in 10 ml 0.01 mM phosphate-buffered saline containing (0.25 M) sucrose and nuclei and large cell debris were removed by centrifugation at 1500 ×g for 10 min. Microsomal and cytosolic fractions were harvested after subsequent centrifugation at 10,000 ×g for 1 h and at 105,000 ×g for 1 h twice. The protein concentrations in cell lysates and subcellular fractions were measured using a kit (number 500-0006, Bio-Rad Laboratories, Inc., Hercules, CA) with bovine serum albumin as a standard. The concentrations of rat's 5*α*-Red1, 3*α*-HSD, and RDH2 proteins were 20 mg/ml. The proteins were used for the measurement of 5*α*-Red1, 3*α*-HSD, and RDH2 activities.

### 2.4. Measurement of 5*α*-Red1, 3*α*-HSD, and RDH2 Activities

5*α*-Red1 activity was measured by incubating 1000 nM testosterone spiked with 60,000 dpm of [^3^H] testosterone as the substrate, 10 *μ*g SRD5A1-containing microsomal protein, and 0.2 mM NADPH in 250 *µ*l PBS (pH = 7.2). 3*α*-HSD activity was measured by incubating 1000 nM dihydrotestosterone spiked with 60,000 dpm of [^3^H]-dihydrotestosterone as the substrate, 10 *μ*g 3*α*-HSD-containing cytosolic protein, and 0.2 mM NADPH in 250 *µ*l PBS (pH = 7.2). RDH2 activity was measured by incubating 1000 nM DIOL spiked with 630,000 dpm of [^3^H] DIOL as the substrate, 10 *μ*g RDH2-containing microsomal protein, and 0.2 mM NAD^+^ in 250 *µ*l PBS (pH = 7.2). 100 *µ*M fungicides were incubated in the respective reaction mixture at 37°C for 60 min for the initial inhibition test. The inhibitory potency of fungicides was measured relative to the control (only DMSO). Each fungicide was dissolved in DMSO and an aliquot (1 *µ*l) of each fungicide was added to the reaction mixture at a final concentration of 0.4%, at which concentration DMSO did not inhibit 5*α*-Red1, 3*α*-HSD, or RDH2 activities. The reaction was stopped with 1 ml ice-cold ether. The steroids were extracted with ether after vigorous vortexing. The organic ether layer was transferred to the new glass tube and dried under nitrogen. The steroids were separated chromatographically on the thin layer plate in chloroform and methanol (90 : 3, v/v), and the radioactivity was measured using a scanning radiometer (System AR2000, Bioscan Inc., Washington, DC) as previously described [14]. The percentage conversion of testosterone into dihydrotestosterone (for 5*α*-Red1), dihydrotestosterone into DIOL (for 3*α*-HSD), and DIOL into dihydrotestosterone (for RDH2) was calculated by dividing the radioactive counts identified as the respective steroids by the total counts of control DMSO.

### 2.5. Determination of Enzyme Kinetics

The enzyme kinetics was determined by adding 0.0315–10 *µ*M testosterone or dihydrotestosterone or DIOL for 5*α*-Red1, 3*α*-HSD, and RDH2. The Michaelis–Menten equation was used by GraphPad (Version 6, GraphPad Software Inc., San Diego, CA) to calculate the apparent Michaelis–Menten constant (*K*_*m*_) and the apparent maximum velocity (*V*_max_). The initial velocity (*V*_*o*_) depends on the apparent *K*_*m*_, *V*_max_, and the substrate concentration ([*S*]) as *V*_*o*_ = *V*_max_[*S*]/(*K*_*m*_ + [*S*]).

### 2.6. Determination of IC_50_ Values and Inhibitory Modes

The half maximum inhibitory concentration (IC_50_) of TEB or TRI to inhibit 5*α*-Red1 was determined by adding 1000 nM of testosterone with 0.2 mM NADPH and 10^−8^–10^−4^ M TEB or TRI in 250 *μ*l phosphate-buffered saline (0.1 mM) containing 5*α*-Red1 protein and incubating each reaction mixture for 60 min. The IC_50_ value of TRI to inhibit 3*α*-HSD was determined by adding 1000 nM of dihydrotestosterone with 0.2 mM NADPH and 10^−8^–10^−4^ M TRI in 250 *μ*l phosphate-buffered saline (0.1 mM) containing 3*α*-HSD protein and incubating each reaction mixture for 60 min. For determining the mode of inhibition of 5*α*-Red1, 10^−9^–10^−5^ M testosterone was added to the reaction mixture in the presence of TEB (10 and 20 *µ*M) or TRI (20 and 40 *µ*M) for 5*α*-Red1. For determining the mode of inhibition of 3*α*-HSD, 10^−9^–10^−5^ M dihydrotestosterone was added to the reaction mixture in the presence of TRI (25 and 50 *µ*M) for 3*α*-HSD.

### 2.7. Preparation of Protein and Ligand Structures and Docking

The crystal structure of rat's 3*α*-HSD containing NADP^+^ and testosterone (PDB id 1afs [15]) was used as a docking target for steroid substrate DIOL, TEB, TRI, and VCZ. These chemical structures were obtained from PubChem (https://pubchem.ncbi.nlm.nih.gov) as ligands. Docking calculations were performed with SwissDock, a docking algorithm based on the docking software EADock DSS [16]. The docked file was visualized using the program Chimera 1.1.1 (San Francisco, CA) and the free energy was calculated.

### 2.8. Statistics

Each experiment was repeated four times. Data were subjected to a nonlinear regression analysis by GraphPad (Version 6, GraphPad Software Inc., San Diego, CA) for IC_50_ values. Lineweaver–Burk plot was used for the mode of inhibition. Data were subjected to an analysis by ANOVA followed by ad hoc Tukey's comparison to identify significant differences between the control (CON) and TEB, TRI, or VCZ group. All data are expressed as means ± SEM. The difference was regarded as significant at *P* < 0.05.

## 3. Results

### 3.1. Effects of Fungicides on 5*α*-Red1

**Figure 1:**
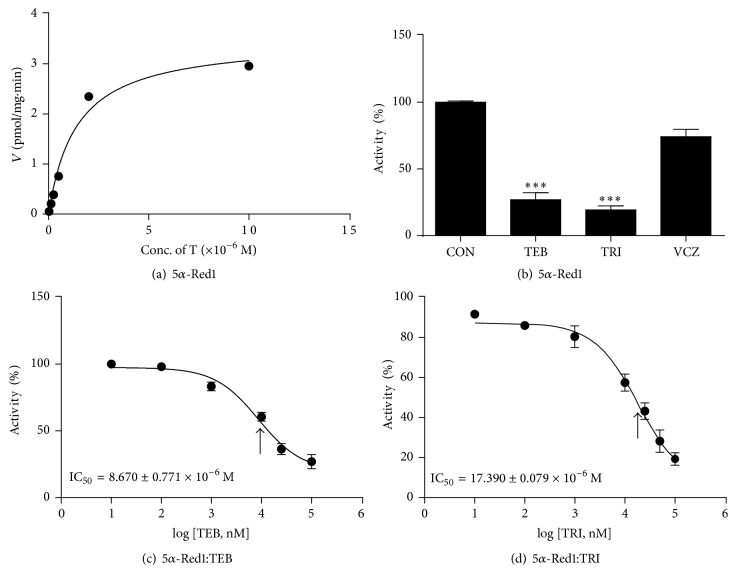
Kinetics of SRD5A1 and the inhibition of fungicides. Panel (a): kinetics of SRD5A1 with testosterone (T) as the substrate. Panel (b): % inhibition by tebuconazole (TEB), triadimefon (TRI), and vinclozolin (VCZ) at 100 *µ*M. Panels (c) and (d): IC_50_ values of TEB and TRI. Mean ± SEM; *∗∗∗* indicates a significant difference compared to the control (CON) at *P* < 0.001.

**Figure 2:**
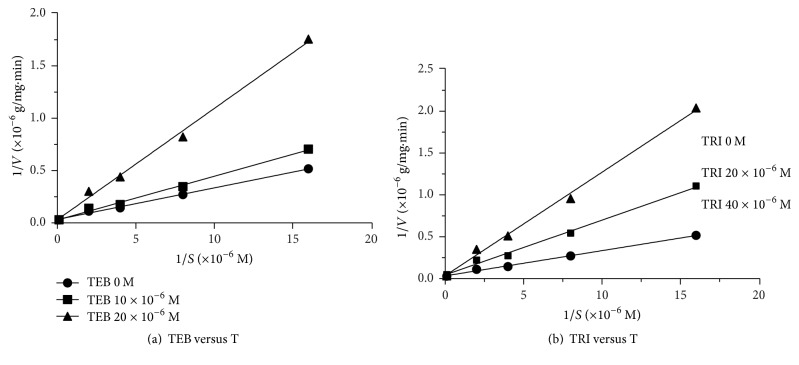
The inhibitory mode of tebuconazole (TEB) and triadimefon (TRI) on rat's SRD5A1. Lineweaver–Burk plots in presence of testosterone and TEB (Panel (a)) as well as testosterone and TRI (Panel (b)). Values were obtained from four samples.

The conversion of testosterone into DHT is catalyzed by 5*α*-Red1, which requires NADPH as a cofactor; the apparent *K*_*m*_ and apparent *V*_max_ of 5*α*-Red1 were 1.397 ± 0.35 *μ*M (mean ± SE, *n* = 4) and 3.494 ± 0.287 pmol dihydrotestosterone/mg protein/min (mean ± SE, *n* = 4), respectively (Table 1 and Figure 1(a)). As presented in Figure 1(b), when the highest concentration (100 *μ*M) was tested, TEB and TRI inhibited rat's 5*α*-Red1 to 26.94 ± 5.30% and 19.31 ± 3.6% of the control value, respectively, but VCZ only to 74.17 ± 5.57% of the control value. We further calculated the IC_50_ values of TEB (Figure 1(c)) and TRI (Figure 1(d)), which were 8.670 ± 0.771 and 17.390 ± 0.079 *μ*M, respectively (Table 1). The modes of inhibition of TEB and TRI on 5*α*-Red1 were found to be competitive against testosterone (Figures 2(a) and 2(b)).

### 3.2. Effects of Fungicides on 3*α*-HSD Activity

**Figure 3:**
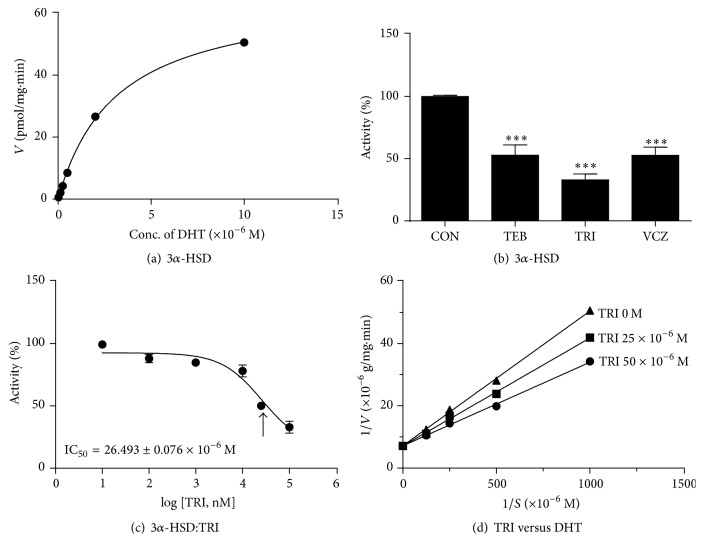
The kinetics of AKR1C14 and the inhibition of fungicides. Panel (a): kinetics of AKR1C14 with dihydrotestosterone (DHT) as the substrate. Panel (b): % inhibition by tebuconazole (TEB), triadimefon (TRI), and vinclozolin (VCZ) at 100 *µ*M. Panel (c): IC_50_ value of TRI. Panel (d): the mode of inhibition of TRI versus DHT. Mean ± SEM; *∗∗∗* indicates a significant difference compared to the control (CON) at *P* < 0.001.

The conversion of dihydrotestosterone into DIOL is catalyzed by 3*α*-HSD, which requires NADPH as a cofactor; the apparent *K*_*m*_ and apparent *V*_max_ of 3*α*-HSD were 3.148 ± 0.197 *μ*M (mean ± SE, *n* = 4) and 66.69 ± 1.587 pmol DIOL/mg protein/min (mean ± SE, *n* = 4), respectively (Table 1 and Figure 3(a)). TRI inhibited rat's 3*α*-HSD to 32.95 ± 4.80% of the control value, while TEB and VCZ caused about 52.78 ± 8.278% and 52.65 ± 6.70% of the control value, respectively (Figure 3(b)). We further calculated the IC_50_ value of TRI, which was 26.493 ± 0.076 *µ*M (Table 1 and Figure 3(c)). The mode of inhibition of TRI on 3*α*-HSD was found to be competitive against dihydrotestosterone (Figure 3(d)).

### 3.3. Effects of Fungicides on RDH2 Activity

**Figure 4:**
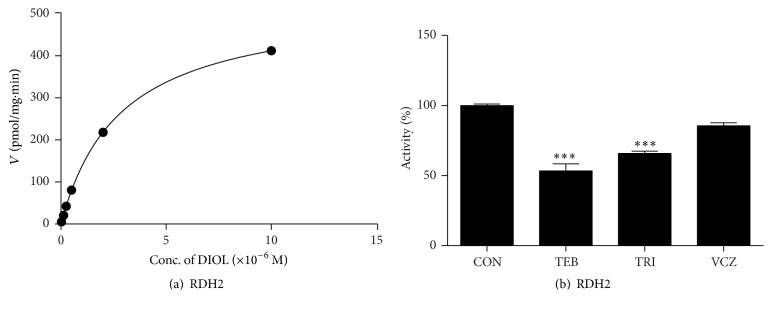
The kinetics of RDH2 and the inhibition of fungicides. Panel (a): kinetics of RDH2 with androstanediol (DIOL) as the substrate. Panel (b): % inhibition by tebuconazole (TEB), triadimefon (TRI), and vinclozolin (VCZ) at 100 *µ*M. Mean ± SEM; *∗∗∗* indicates a significant difference compared to the control at *P* < 0.001.

The conversion of DIOL into dihydrotestosterone is catalyzed by RDH2, which requires NAD^+^ as a cofactor; the apparent *K*_*m*_ and apparent *V*_max_ of RDH2 were 2.850 ± 0.037 *μ*M (mean ± SE, *n* = 4) and 529.5 ± 2.612 pmol dihydrotestosterone/mg protein/min (mean ± SE, *n* = 4), respectively (Table 1 and Figure 4(a)). TRI and TEB only inhibited RDH2 to 65.79 ± 1.69% and 53.35 ± 5.03% of the control value, while VCZ did not inhibit the enzyme activity (85.51 ± 2.20% of the control value, Figure 4(b)).

### 3.4. Docking of Fungicides to 3*α*-HSD

**Figure 5:**
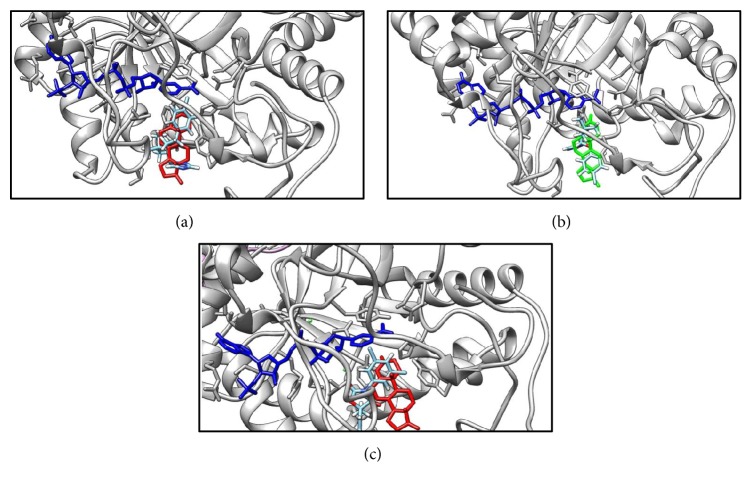
Docking analysis for the binding to rat's AKR1C14 (1AFS). Panel (a): tebuconazole; blue structure, NADPH; red structure, testosterone; sky-blue structure, tebuconazole. Panel (b): triadimefon; blue structure, NADPH; green structure, dihydrotestosterone; sky-blue structure, triadimefon. Panel (c): vinclozolin; blue structure, NADPH; red structure, testosterone; sky-blue structure, vinclozolin.

**Figure 6:**
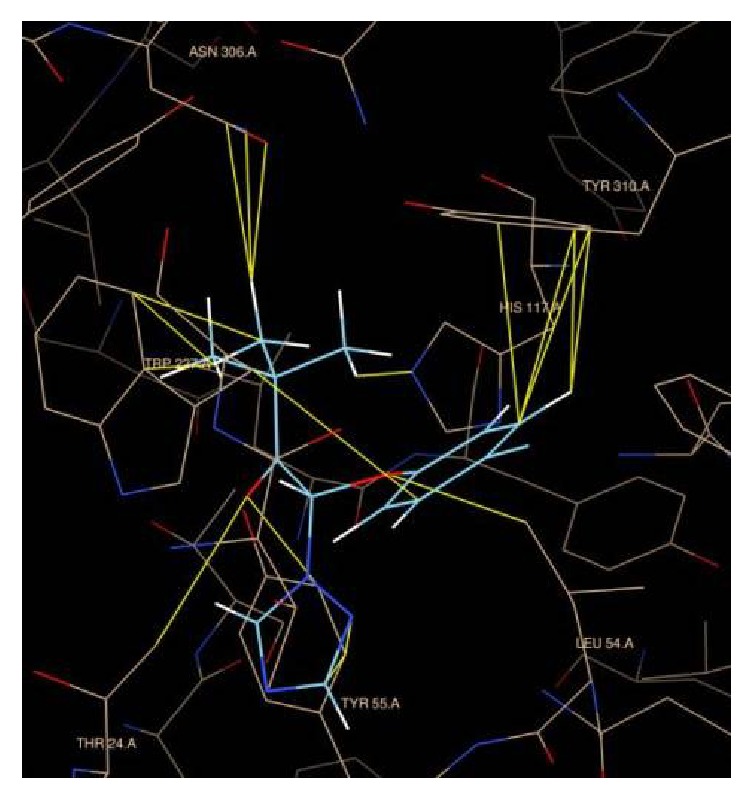
Docking analysis for the binding of triadimefon to rat's AKR1C14 (1AFS). The residues of AKR1C14 interacting with triadimefon were listed.

Because among three enzymes only the crystal structure of rat's 3*α*-HSD is available, we docked DIOL to 3*α*-HSD first. DIOL was found to bind to the dihydrotestosterone-binding pocket, with free energy of −7.73 Kcal. Further docking analysis for TEB (Figure 5(a)), TRI (Figure 5(b)), and VCZ (Figure 5(c)) showed that all these three chemicals bound to the steroid-binding pocket, with free energies of 7.28, −7.63, and −7.34. These data indicate that TRI has the highest binding affinity with 3*α*-HSD. TRI interacts with Try310, Trp227, His117, Tyr55, Leu54, Thr24, and Asn306 residues of 3*α*-HSD (Figure 6). The Tyr310 and Trp227 residues were believed to hold the steroid structure, and His117 and Tyr55 residues were believed to catalyze the 3*α*-position of the steroid [15].

## 4. Discussion

In the brain, the neurosteroidogenic enzymes 5*α*-Red1 [17], 3*α*-HSD [11, 17], and RDH2 [12] are involved in the biosynthesis and metabolism of neurosteroids. 5*α*-Red1 and 3*α*-HSD are responsible for the neurosteroid biosynthesis to form 3*α*-reduced neurosteroids, while RDH2 is responsible for the neurosteroid metabolism to remove the 3*α*-reduced neurosteroids. These neurosteroidogenic enzymes showed different sensitivity to some fungicides. Here, we demonstrated that TEB and TRI potently inhibited 5*α*-Red1, the irreversible step of neurosteroid biosynthesis. Furthermore, TRI also potently inhibited 3*α*-HSD, thus leading to the reduced level of neurosteroids. VCZ was the weakest fungicide to inhibit 5*α*-Red1 and 3*α*-HSD.

Interestingly, the enzyme 5*α*-Red1 is the most sensitive to the inhibition by TEB compared to 3*α*-HSD and RDH2. The IC_50_ values of TEB for 5*α*-Red1, 3*α*-HSD, and RDH2 were 8.67, ~100, and ~100 *µ*M. 5*α*-Red1 and 3*α*-HSD share equal sensitivity to the inhibition by TRI compared to RDH2. The IC_50_ values of TRI for 5*α*-Red1, 3*α*-HSD, and RDH2 were 17.39, 26.49, and ~100 *µ*M. The reason for this difference is still unclear. This is possibly due to the difference of these enzyme structures. 5*α*-Red1 is the rate-limiting irreversible step for the formation of many neurosteroids. Animal study suggests subsequent 3*α*-reduction of dihydroprogesterone and dihydrotestosterone by 3*α*-HSD into steroid metabolites which have neuroactive function via enhancing GABA suppression. These neuroactive steroids promote GABA effects by allosteric modulation at GABA-A receptors, thus exerting anticonvulsant, antidepressant, and anxiolytic effects [18]. In socially isolated mice, 5*α*-Red1 is downregulated in glutamatergic pyramidal neurons that converge on the amygdala from cortical and hippocampal regions possibly causing anxiety, aggression, and cognitive dysfunction [19, 20].

VCZ was the weakest inhibitor for 5*α*-Red1 and 3*α*-HSD, with IC_50_ about 100 *µ*M. However, VCZ almost did not inhibit RDH2 when 100 *µ*M was used. The reason why the potency of VCZ is different from those of TEB and TRI is unclear. This is possibly due to the different chemical structures, in which TEB and TRI contain one triazole and VCZ contains one imidazole in the chemical structure.

TEB and TRI competitively inhibited 5*α*-Red1 when testosterone was provided. TEB and TRI also competitively inhibited 3*α*-HSD. Docking study further confirmed that these three chemicals bound to the steroid-binding pocket of 3*α*-HSD. TRI interacts with Try310, Trp227, His117, Tyr55, Leu54, Thr24, and Asn306 residues in the steroid-binding pocket of 3*α*-HSD. The Tyr310 and Trp227 residues were believed to maintain stability of the steroid, and His117 and Tyr55 residues of 3*α*-HSD were believed to catalyze the 3*α*-position of the steroid [15]. The free energy calculation further showed the lowest binding energy for TRI, which was comparable to DIOL, indicating that TRI has high affinity for 3*α*-HSD.

The homeostasis of neurosteroids including ALLO and DIOL depends on the catalysis of their biosynthetic enzymes, 5*α*-Red1 and 3*α*-HSD, as well as the metabolizing enzyme RDH2. Since 5*α*-Red1 is the rate-limiting step for neurosteroid formation, this inhibition by TEB and TRI is critical for the production of neurosteroids. Indeed, evidence shows that these fungicides can affect brain function. Rats after exposure to triadimefon developed a deficit in spatial learning and reference memory [21]. Rats after perinatal exposure to tebuconazole produced neurobehavioral deficits and neuropathology [22]. Triadimefon also disrupted the transporter of extracellular dopamine, dihydroxyphenylacetic acid, homovanillic acid, and 5-hydroxyindoleacetic acid in adult rat's striatum [23]. Goldfish after acute and chronic exposure to VCZ developed dysfunction of neuroendocrine regulation of reproduction [24]. Therefore, the disruption of neurosteroid biosynthesis by these fungicides could lead to neurological dysfunction.

In conclusion, TEB and TRI are inhibitors of 5*α*-Red1 and 3*α*-HSD. TEB inhibited 5*α*-Red1 activity more potently than the activities of 3*α*-HSD and RDH2. Their negative effects on the neurosteroid accumulation were worthy of further research.

## Figures and Tables

**Table 1:** The enzyme kinetic parameters and the half maximal inhibitory concentration (IC_50_) of fungicides.

Parameters	5*α*-Red1	3*α*-HSD	RDH2
Apparent *K*_*m*_ (*µ*M)	1.397 ± 0.35	3.148 ± 0.197	2.850 ± 0.037
Apparent *V*_max_ (pmol/mg·min)	3.494 ± 0.287	66.69 ± 1.589	529.5 ± 2.612
IC_50_ (*µ*M)			
Tebuconazole	8.670 ± 0.771	~100	>100
Triadimefon	17.39 ± 0.079	26.493 ± 0.076	>100
Vinclozolin	NI	~100	NI

Mean ± SE, *n* = 4. NI: no inhibition at 100 *µ*M.

## Abbreviations

3*α*-HSD: 3*α*-Hydroxysteroid dehydrogenase
ALLO: Allopregnanolone
DHT: Dihydrotestosterone
DIOL: Androstanediol
IC_50_: Half maximum inhibitory concentrations
RDH2: Retinol dehydrogenase 2
5*α*-Red1: 5*α*-Reductase 1
TEB: Tebuconazole
T: Testosterone
TRI: Triadimefon
VCZ: Vinclozolin.
